# A Novel Lightweight Mechanical Metamaterial with a Tunable Thermal Expansion Coefficient

**DOI:** 10.3390/ma18081761

**Published:** 2025-04-11

**Authors:** Zhedong Xie, Bing Tian, Yingbo Li, Chao Zhang, Yuxuan Liu, Hongyu Guo

**Affiliations:** College of Engineering and Technology, Jilin Agricultural University, Changchun 130118, China; xiezhedong@126.com (Z.X.); 18732602180@163.com (B.T.); liyingboybl@126.com (Y.L.); zcsuper123456@163.com (C.Z.); hglyx97_315@163.com (Y.L.)

**Keywords:** tunable coefficient of thermal expansion, negative thermal expansion, mechanical metamaterial, lightweight

## Abstract

In natural materials, thermal expansion is typically positive, and negative thermal expansion is rarely observed. The tunable thermal expansion properties of mechanical metamaterials offer a promising solution to challenges caused by rapid temperature fluctuations. Therefore, this study proposes a dual-material double-trapezoidal hexagonal mechanical metamaterial (DTH), and derives the thermoelastic equations that build the relationship between temperature, external force, and displacement. Through theoretical analysis and numerical simulation, the intrinsic mechanism between the CTE and geometric parameters of DTH is revealed. Through the synergistic effect of dual materials and structural design, this metamaterial not only achieves thermal expansion regulation but also enhanced lightweight performance. The results show that by controlling the geometric parameters of DTH, the adjustment of effective CTE and elastic modulus can be realized, and the metamaterial composed of positive CTE materials can achieve a range of thermal expansion behaviors, including near-zero CTE and negative CTE. The tunable thermal expansion range extends from +39.92 ppm/°C to −3640.6191 ppm/°C. The metamaterials proposed in this study are not only superior to traditional materials in terms of thermal expansion performance but also have the characteristics of light weight and simple structure. This multifunctional material achieves higher performance and adaptability in applications.

## 1. Introduction

Mechanical metamaterials, also known as mechanical or solid-state metamaterials, exhibit extraordinary mechanical behaviors [1]. Qualities such as negative Poisson’s ratio (NPR) [2,3,4,5], negative thermal expansion (NTE) or zero thermal expansion (ZTE) [6,7,8,9,10], and high stiffness [11,12,13,14,15] are among the other significant properties. These unusual material properties are primarily controlled by microgeometry rather than traditional base materials [1], thus providing a wider range of options for designing metamaterial structures that are superior to conventional materials.

In nature, most common materials exhibit positive thermal expansion, following the principle of thermal expansion and cold contraction. Materials with negative thermal expansion properties are extremely rare. For instance, the negative thermal expansion property of Hg_2_I_2_ [16] is attributed to its unique crystal structure and the mechanism of mercury–mercury bonding. However, the toxicity and chemical reactivity of Hg_2_I_2_ significantly limit its practical applications. The negative thermal expansion property of zeolites [17] is primarily caused by the contraction of their porous structure. Zirconium tungstate (ZrW_2_O_8_) exhibits negative thermal expansion properties due to the transverse vibrations of its structural framework [18]. The above materials can only be manifested as NTE within a specific and narrow temperature range, which is greatly limited to the scope of application. When instruments operate in environments with drastic temperature fluctuations, the thermal deformation of conventional materials can affect manufacturing precision and even lead to mechanical failure. For example, structures in fields such as precision instruments and aerospace are prone to experiencing significant temperature fluctuations, which can lead to adverse thermal deformation [19,20]. Therefore, metamaterials with ZTE properties are highly suitable for applications where thermal deformation must be minimized or eliminated. Zero thermal expansion (ZTE) properties can be achieved through the combined effects of specific negative thermal expansion (NTE) materials and positive thermal expansion (PTE) materials to meet the requirements of thermal expansion compensation [21,22]. Alternatively, ZTE can be directly obtained using mechanical metamaterials with tunable thermal expansion coefficients (CTEs). Furthermore, NTE properties can also be obtained through mechanical metamaterials with tunable CTEs. Therefore, the urgent need for mechanical metamaterials with a wide range of tunable CTEs to adapt to different application environments and mitigate the adverse effects caused by temperature fluctuations [23,24,25] is one of the keys to the research in this paper.

Tunable-CTE mechanical metamaterials, as a type of mechanical metamaterial, exhibit tunable thermal expansion properties that are closely related to their artificially designed unit cell structures. The design mechanism involves combining different constituent materials, whose interactions and coordinated thermal deformations regulate the overall thermal expansion of the structure. Therefore, the study of metamaterials can be converted into the design and analysis of their unit cell structures. Based on their deformation characteristics when undergoing thermal deformation, they can be classified into two categories: the bending-dominated type [26,27,28] and the stretching-dominated type [12,29,30,31,32,33,34,35]. Compared to bending-dominated metamaterials, stretching-dominated metamaterials exhibit higher stiffness due to their inherent stretching-dominated deformation [36]. The stiffness characteristics play a crucial role in determining the response of lightweight mechanical metamaterials in thermal environments. In stretching-dominated metamaterials, NTE is achieved through the differences in thermal expansion between the constituent components. The mismatch in thermal expansion between dual materials is an effective method for modulating the CTE [37]. Miller [38] proposed a conceptual model that established a rational design framework for NTE structures and designed bi-material triangular structures with zero and negative CTEs. Wei et al. [39,40,41,42] conducted extensive studies on stretching-dominated bi-material triangular NTE metamaterials and extended the research to three-dimensional structures, advancing the development of NTE metamaterials. Yu et al. [43] proposed a chiral lattice composite structure with a wide range of tunable CTEs by leveraging the bending of bi-material beams and the unique deformation mechanisms of chiral lattice structures. Inspired by traditional bi-material triangular structures, Ye et al. [44] proposed a novel bi-material mechanical metamaterial structure. By introducing additional geometric variables, this structure achieved a broader range of tunable CTEs. Inspired by the Hoberman sphere, Li et al. [45,46] proposed a rhombus structure composed of two bi-material triangles. Multiple rhombuses arranged in a circular array form a circular metamaterial unit cell and can achieve zero and negative thermal expansion behaviors. In addition, lightweight characteristics are a crucial objective for many engineering applications, particularly in the aerospace field. The National Aeronautics and Space Administration (NASA) has identified lightweighting as one of the key technologies for development between 2010 and 2030. The study by Chen et al. demonstrated that by carefully tuning unit cell geometry and base materials, mechanical metamaterials can simultaneously achieve a low thermal expansion coefficient (CTE), high stiffness, high strength, and lightweight properties, making them suitable for various engineering applications [41]. Therefore, in addition to achieving tunable thermal expansion properties, material and structural designs must also incorporate lightweight considerations.

Although numerous metamaterials have been developed to regulate thermal expansion, their thermal expansion behavior often involves complex thermoelastic effects, which are significantly influenced by microstructural features and interfacial heat transfer characteristics. To optimize metamaterial design, precise measurement of their thermoelastic properties is essential. In 2007, Tomoda et al. investigated nanoscale thermal diffusion and thermoelastic behavior and proposed a high-precision thermal measurement technique, providing valuable insights for understanding and optimizing the thermoelastic properties of metamaterials [47]. In 2009, Dehoux et al. further utilized ultrafast optical techniques to measure heat flux across metamaterial interfaces, offering theoretical support for understanding the thermoelastic behavior of structural materials [48]. In 2015, Matsuda et al. introduced a method based on ultrashort laser pulses to excite and detect ultra-high-frequency acoustic waves within materials, enabling characterization of their elastic properties and thermal expansion behavior [49]. This technique provides a precise experimental approach for measuring the thermoelastic properties of negative-thermal-expansion mechanical metamaterials, facilitating the validation of theoretical models and advancing their practical applications. However, it is noteworthy that most existing tunable thermal expansion metamaterials are designed based on triangular unit structures, with relatively limited research focusing on their lightweight characteristics. Therefore, developing metamaterials that combine tunable thermal expansion with lightweight properties is of fundamental and pioneering significance for engineering applications.

Here, this study proposes a lightweight double-trapezoidal hexagonal bi-material metamaterial (DTH) composed of Nylon and PVA. Based on the 2D beam element theory, an elastic model under the coupled effects of temperature and External force loads were developed. The relationships between the material’s CTE, elastic modulus, relative density, and geometrical parameters were comprehensively analyzed. A series of parametric analyses were conducted to identify geometrical structures exhibiting zero and negative CTEs. Finally, the results of finite element numerical simulations were compared with theoretical predictions, demonstrating excellent agreement. These models demonstrate how adjusting geometric parameters can be used to design structures with NTE or ZTE, thereby providing an effective approach for analyzing and designing other structures with tunable thermal expansion coefficients. These mechanical metamaterials with tunable CTEs and lightweight characteristics are well suited for high-tech fields requiring high performance and multifunctionality, while also offering new options for material upgrades in traditional industries. For instance, they can be applied to critical structural components of aircraft, satellites, rockets, and other spacecrafts. Such applications not only enhance stability control in engineering structures under thermal environments, ensuring longer service life, but also achieve lightweight performance, reducing fuel consumption and launch costs.

## 2. Materials and Methods

### 2.1. Design of Metamaterials

**Figure 1:**
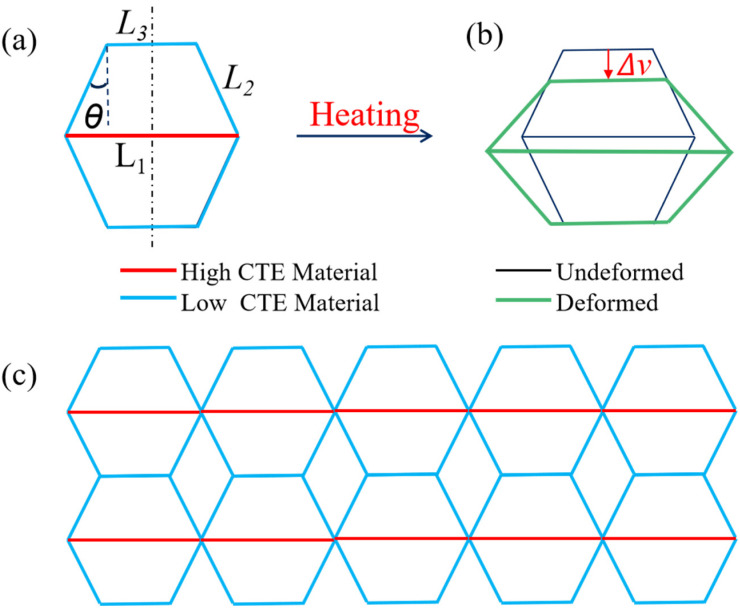
The theoretical model of thermal deformation for the DTH. (**a**) The unit cell before heating, (**b**) the unit cell after heating, where ∆v  represents the thermal displacement variation in the y-direction; (**c**) the planar metamaterial constructed from DTH unit cells.

The intrinsic mechanism of tunable CTEs in metamaterials lies in the architectural design of the bi-material hexagonal unit cell. The difference in CTE between the constituent materials is the key to designing NTE mechanical metamaterials. This study introduces a double-trapezoidal hexagonal mechanical metamaterial (DTH), whose unit cell consists of two identical isosceles trapezoids connected by a bottom beam, forming a hexagonal structure, as illustrated in Figure 1. The geometric characteristics of the metamaterial are defined by the following parameters: bottom beam length $L_{1}$, inclined beam length $L_{2}$, top beam length $L_{3}$, inclination angle *θ*, and the length ratio λ, where λ = $L_{3}$/$L_{1}$ (top beam/bottom beam). The DTH metamaterial is composed of Nylon and PVA, two materials with distinct CTEs. Figure 1a illustrates the DTH cell structure before heating. In this structure, the lower base beam $L_{1}$ (red rod) is composed of Nylon, while the upper base beam $L_{3}$ and the two diagonal beams $L_{2}$ (blue rods) are made of PVA. θ is the characteristic angle between the diagonal beams and the normal line perpendicular to the upper base beam. All beams have an equal cross-sectional thickness  t in the in-plane direction and share the same cross-sectional width b in the out-of-plane direction. Figure 1b shows the DTH unit cell structure after heating. Ideally, the contraction of the structure in the height direction upon elevated ambient temperature reflects the negative thermal expansion property in the height direction; that is, ΔH < 0. Figure 1c illustrates the planar metamaterial composed of an array of DTH cell structures. Under elevated ambient temperatures, the planar metamaterial exhibits the same height-direction thermal contraction behavior as the heated DTH unit cell structure. 

It is worth noting that the structural parameters of the DTH are required as follows: 0 < θ < 90° and 0 < λ < 1. When θ = 0°, the structure degenerates into a quadrilateral, and when θ = 90°, the beams overlap, failing to form a valid structure. Therefore, the condition 0° < θ < 90° is required. To facilitate dimensionless analysis and simplify parameter evaluation, the length ratio between the top and bottom beams is defined as λ = L3 L1, with a valid range of 0 < λ< 1. Based on Figure 1, the length of the inclined beam is given by $L_{2}=\frac{L_{1}\left(1-\lambda \right)}{2\sin\theta }$. For consistency in this study, the bottom beam length $L_{1}$ is set as a fixed value of 100 mm, unless otherwise stated.

### 2.2. Analytical Model

The stiffness matrix method in structural mechanics analysis is used to derive the relationship between force and deformation in mechanical metamaterials, forming the basis for studying the mechanical properties of unit cell structures, such as CTE and elastic modulus. Therefore, in this study, a stiffness matrix was constructed to simulate the mechanical behavior of the structure, aiming to address the key issues between force and deformation in the structure.

**Figure 2:**
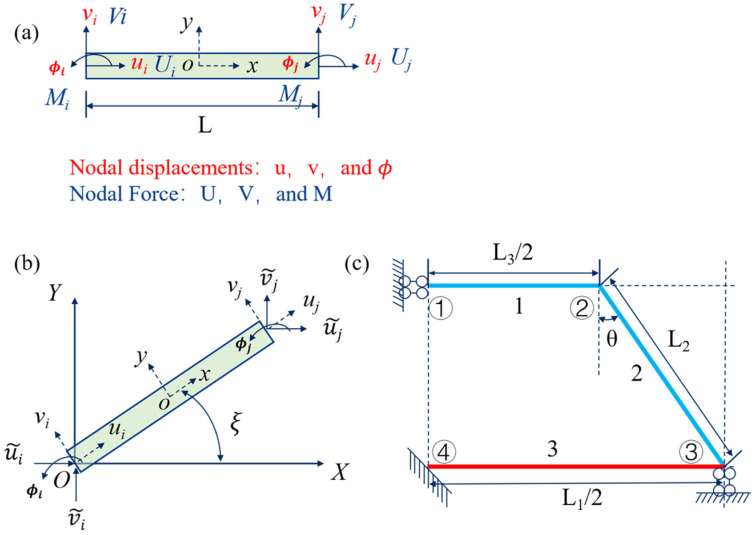
The planar beam element and 1/4 unit cell structure framework with boundary conditions. (**a**) Beam element in the local coordinate system, showing nodal displacements, rotations, forces, and moments. (**b**) Transformation from the local coordinate system to the global coordinate system. (**c**) Nodes and boundary constraints of 1/4 unit cell structure. The beam elements are labeled as 1, 2, and 3, where beams 1 and 2 (in blue) are made of PVA, and beam 3 (in red) is made of Nylon. The nodes are labeled as ①, ②, ③, and ④.

Due to the symmetry of the DTH metamaterial structure, a quarter of the 2D cell structure was used in this study as the base model for the analysis of the structure, which was used to estimate the relevant mechanical properties of the proposed mechanical metamaterial. In this case, the element and node numbering, as well as the boundary conditions of the quarter DTH unit cell structure, are shown in Figure 2. Due to the symmetry of the structure, it can be inferred that any External force applied at the boundary of the quarter structure will be symmetrically distributed across the remaining parts of the structure. Consequently, there are no effective bending forces, torques, or shear forces present at the boundaries of the analytical model.

For a planar beam element, the general elastic equation relating nodal forces and nodal displacements can be expressed as Equation (1):

(1) $$\left\{F\right\}={\left[k\right]}^{e}\left\{\delta \right\},$$

Here, the nodal forces and nodal moments are denoted as ${\left\{U_{i}\;V_{i}\;M_{i}\right\}}^{T}$ and ${\left\{U_{j}\;V_{j}\;M_{j}\right\}}^{T}$, while the nodal displacements and nodal rotations are represented as ${\left\{u_{i}\;v_{i}\;\varphi_{i}\right\}}^{T}$ and ${\left\{u_{j}\;v_{j}\;\varphi_{j}\right\}}^{T}$, respectively. Specifically, the nodal forces for the element are represented as $\left\{F\right\}={\left\{{U_{i}\;V_{i}\;M_{i}\;U}_{j}\;V_{j}\;M_{j}\right\}}^{T}$, and the nodal displacements as $\left\{\delta \right\}={\left\{u_{i}\;v_{i}\;\varphi_{i\;}u_{j}\;v_{j}\;\varphi_{j}\right\}}^{T}$, as shown in Figure 2a. Among them, ${\left[k\right]}^{e}$ represents the local stiffness matrix of the element, with its detailed expression provided in Appendix A Equation (A1).

Based on the thermal expansion formula in Equation (2), the strain of the beam element can be determined, as expressed in Equation (3):

(2) $$\alpha =\frac{∆L}{L∆T\;},$$

(3) ε=α∆T,

The local thermal load for the beam element is given in Equation (4), and its vector form is expressed in Equation (5):

(4) $$K_{T}=EA\alpha ∆T\;,$$

(5) $${\left\{K_{T}\right\}}^{e}={\left[\begin{array}{cccccc} EA\alpha ∆T & 0 & 0 & -EA\alpha ∆T & 0 & 0 \end{array}\right]}^{T},$$

To facilitate the analysis, it is necessary to transform the load and displacement vectors from the local coordinate system (x-y) to the global coordinate system (X-Y). As shown in Figure 2b, during the transformation from the local to the global coordinate system, the element’s global stiffness matrix and the element’s global thermal load are expressed as Equations (6) and (7).

(6) $${\left[K\right]}^{g}={\left[h\right]}^{T}{\left[k\right]}^{e}\left[h\right],$$

(7) $${\left\{K_{T}\right\}}^{g}={\left[h\right]}^{T}{\left\{K_{T}\right\}}^{e},$$

where  [h]  represents the coordinate transformation matrix; its detailed expression can be found in Appendix A Equation (A2).

As shown in Figure 2c, partial boundary conditions can be established. These include allowing node A to move along the *X*-axis, allowing node B to move freely, allowing node C to move along the *Y*-axis, and fully constraining node O. The rotational angles of all the above nodes are set to 0.

Through coordinate transformation, the force-displacement relationship of the beam elements in the local coordinate system is converted into the global coordinate system. By assembling the global stiffness matrix according to the sequence of the node labels, we eventually obtain the thermoelastic Equation (8).

(8) $$\left\{P\right\}={\left[K\right]}^{g}\left\{\delta \right\}-{\left\{K_{T}\right\}}^{g}\;,$$

After applying the boundary conditions, a simplified thermoelastic model is obtained, which represents the thermoelastic model of the 2D metamaterial under the coupled effects of temperature and external mechanical loads. The detailed expression can be found in Appendix A Equation (A6).

When the structure is subjected to temperature changes only, without any external forces, [36] the effective thermal expansion coefficients $\alpha_{x}\;$ and $\alpha_{y\;}$ of the metamaterial can be determined using Equations (9) and (10). In Equation (9), ∆u  represents the thermal displacement change in the x-direction, while ${\bar{u}}_{2}$ corresponds to the experimentally obtained displacement variation in the x-direction. L denotes the original length in the x-direction, which is $L_{1}$. In Equation (10), ∆v represents the thermal displacement change in the y-direction, while ${\bar{v}}_{1}$ corresponds to the experimentally obtained displacement variation in the y-direction. H denotes the original height in the y-direction, ∆T represents the temperature variation.

(9) $$\alpha_{x}=\frac{∆u}{L∆T}=\frac{{\bar{u}}_{2}}{L_{1}∆T}\;,$$

(10) $$\alpha_{y}=\frac{∆v}{H∆T}=\frac{2{\bar{v}}_{1}\sin\theta }{∆TL_{1}\left(1-\lambda \right)\cos\theta },$$

### 2.3. Finite Element Simulations

To validate the theoretical model and gain a better understanding of the proposed metamaterial’s properties, numerical simulations of its thermal response were conducted using the finite element software Abaqus/CAE 2023 (Dassault Systèmes, version 2023, Providence, RI, USA). Here, mechanical performance simulations of the complete 2D metamaterial unit cell structure (Figure 1a) are conducted, focusing primarily on the simulation of the coefficient of thermal expansion (CTE) and elastic modulus. These two performance simulations share three common steps: Firstly, in the Part module, create a 2D geometric model where the components are modeled using beam elements. Secondly, in the Property module, the material properties of the beams are defined, including the elastic modulus, density, and CTE [50]. A tunable thermal expansion metamaterial composed of Nylon and polyvinyl alcohol (PVA) can be easily fabricated using additive manufacturing technology. Therefore, the model uses a bi-material design, where Material 1 is Nylon and Material 2 is PVA. The relevant physical properties are listed in Table 1. Finally, the defined material properties are assigned to the corresponding beams, and the beam cross-sections and orientations are specified. It should be noted that the complete cell structure is symmetrical from the quarter-cell elements. Therefore, the middle beam (the red lower base beam in Figure 1a) needs to have a cross-sectional width twice that of the other beams. The cross-section of the other beams is defined (2 mm × 3 mm), while the cross-section of the middle beam is defined (2 mm × 6 mm). In the Mesh module, mesh the created lattice model with a global size of 1 mm to ensure sufficient mesh quality. The element type is selected as B31 beam elements.

## 3. Results and Discussion

### 3.1. Relative Density

**Figure 3:**
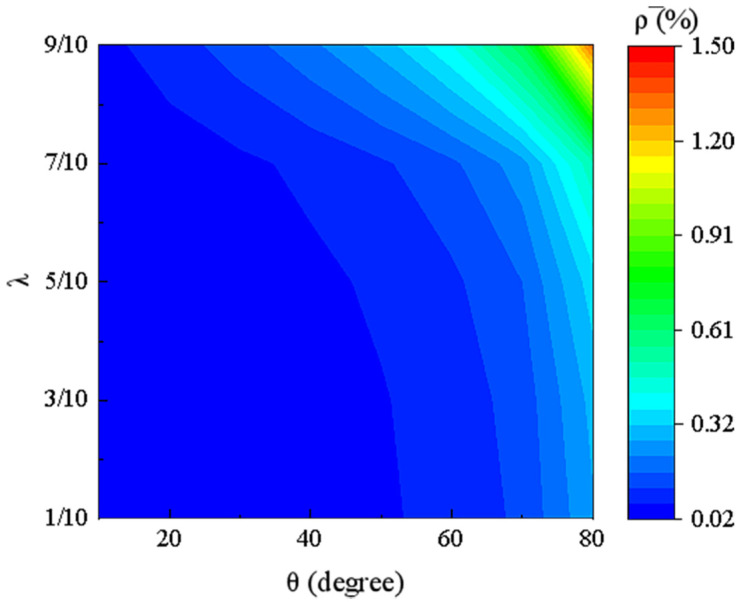
The contour plot of the effective relative density of the double-trapezoidal hexagonal metamaterial (DTH) with respect to the angle θ and the length ratio λ.

The relative density represents the lightweight properties of the material, this can be measured by calculating the relative density of the metamaterial to measure the lightweight properties of the metamaterial structure. The effective density of the metamaterial can be obtained from the mass per unit area. The relative density $\bar{\rho }$ is the ratio of the mass density ρ to the effective density $\rho_{2}$ of the lattice beams [36]. Therefore, the relative density of DTH can be expressed by Equation (11):

(11) $$\bar{\rho }=\frac{\rho }{\rho_{2}}=\frac{24\sin\theta \left[\rho_{1}L_{1}+\rho_{2}\left(\frac{L_{1}\left(1-\lambda \right)}{\sin\theta }+L_{1}\lambda \right)\right]}{\cos\theta \rho_{2}L_{1}^{2}\left(1-\lambda^{2}\right)}\;,$$

where $L_{1}$ and $\rho_{1}$ represent the length and density of Material 1 (Nylon), while $\rho_{2}$ represents the density of Material 2 (PVA). The relationship between relative density and the geometric parameters (θ, λ) is well explained by Equation (14). The variation of the effective relative density of the DTH metamaterial with respect to the angle θ and the length ratio λ can be visualized using the contour plot shown in Figure 3.

As shown in Figure 3, an increase in the angle θ  and the length ratio λ leads to an increase in the relative density. This is because the length of the $L_{3}$ beam increases, resulting in a higher structural mass. Smaller values of the length ratio λ and angle θ are more favorable for lightweight structural design. Therefore, it can be concluded that by reasonably selecting smaller geometric parameters such as θ and λ, the double-trapezoidal hexagonal metamaterial (DTH) can achieve a lower relative density. In addition, it can be observed that the relative density of the double-trapezoidal hexagonal metamaterial (DTH) is significantly less than 2%. This indicates that within the range of λ from 1/10 to 9/10 and θ from 10° to 80°, the DTH metamaterial exhibits excellent lightweight performance.

### 3.2. Coefficient of Thermal Expansion

To simulate the thermal deformation process of the DTH metamaterial unit cell structure, the static analysis step of the Abaqus/CAE 2023 (Dassault Systèmes, version 2023, Providence, RI, USA) was used. By applying a predefined temperature field in the Load module, the initial temperature was set to 20 °C, which was then increased to 60 °C, corresponding to a temperature variation of 40 °C. The boundary condition of the metamaterial unit cell structure is set such that the center point of the bottom edge is fixed, with no other constraints applied. The change in ambient temperature causes the structure to deform along the x and y axes. Therefore, a simulation is performed to study the deformation properties of the structure under elevated ambient temperatures. By calculating the displacement difference Δu  between the left and right nodes of the double-trapezoidal hexagonal unit cell structure, as well as the displacement difference Δv between the two nodes at the middle positions of the upper and lower beams, ref. [36] the effective thermal expansion coefficient of the structure can be determined using the following thermal expansion Equations (12) and (13).

(12) $$\alpha_{x}=\frac{\Delta u}{L\Delta T}\;\;,$$

(13) $$\alpha_{y}=\frac{\Delta v}{H\Delta T}\;\;,$$

Here, Δu represents the horizontal change of the unit during the temperature variation, Δv  represents the vertical change of the unit during the temperature variation, L is the initial length of the unit, H  is the initial height of the unit, and ΔT  is the temperature change.

**Figure 4:**
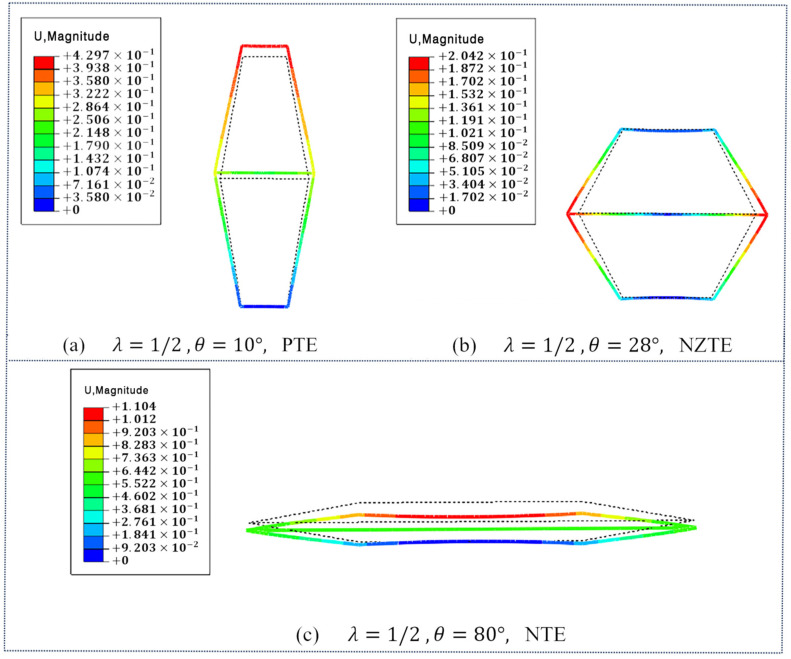
When λ = 1/2, the simulation results of the effective thermal expansion coefficient of the DTH metamaterial in the y-direction under different angles θ: (**a**) at θ = 10°, the material exhibits positive thermal expansion behavior; (**b**) at θ = 28°, the material exhibits near-zero thermal expansion behavior; (**c**) at θ = 80°, the material exhibits negative thermal expansion behavior.

Note that calculations show the metamaterial exhibits positive thermal expansion in the horizontal direction (x-direction) and does not demonstrate anomalous properties. Therefore, it is not the focus of this study. The following discussion will focus solely on the effective thermal expansion coefficient in the y-direction. To study the influence of the length ratio λ  and the angle θ on the thermal expansion coefficient of the DTH metamaterial, two scenarios were considered: (1) fixing λ = 1/2 and varying θ  within the range of 10° to 80°, and (2) fixing θ = 30° and varying λ with values of 1/10, 3/10, 1/2, 7/10, and 9/10. As shown in Figure 4, the simulation results of the effective coefficient of thermal expansion in y-direction for DTH metamaterials at different angles θ are fixed for λ = 1/2. As shown in Figure 4a, when θ = 10°, the DTH metamaterial exhibits thermal expansion in the y-direction upon the ambient temperature rising to 60°C, indicating positive thermal expansion behavior. As shown in Figure 4b, when θ = 28°, the DTH metamaterial shows almost no expansion or contraction in the y-direction under the same temperature increase, with $\alpha_{y}$ = 0.0567 ppm/°C, indicating near-zero thermal expansion behavior. As shown in Figure 4c, when θ = 80°, the DTH metamaterial exhibits thermal contraction in the y-direction upon the temperature rising to 60 °C, indicating negative thermal expansion behavior. Therefore, the results indicate that with the length ratio λ = 1/2 fixed, the effective thermal expansion coefficient $\alpha_{y}$ of the DTH metamaterial in the y-direction can be adjusted between positive, near-zero, and negative values by modifying the angle θ.

**Figure 5:**
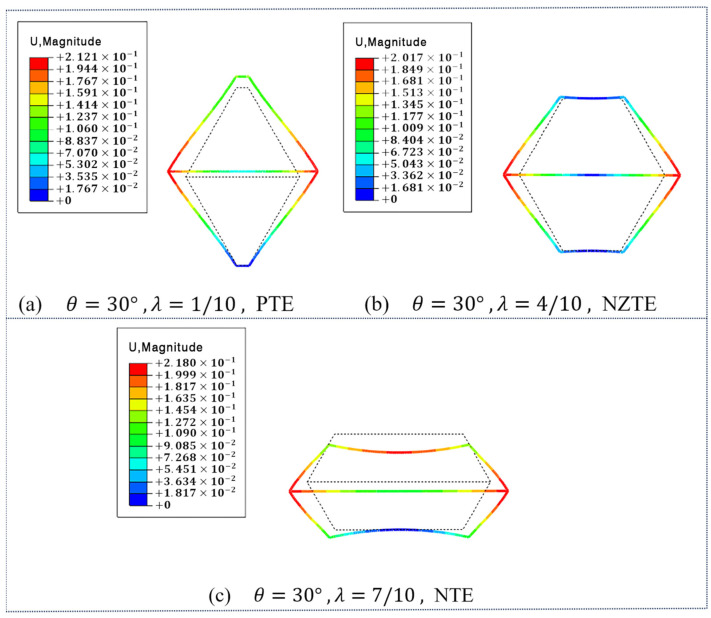
When θ  = 30°, the simulation results of the effective thermal expansion coefficient of the DTH metamaterial in the y-direction under different length ratio λ: (**a**) at λ = 1/10, the material exhibits positive thermal expansion behavior; (**b**) at λ = 4/10, the material exhibits near-zero thermal expansion behavior; (**c**) at λ = 9/10, the material exhibits negative thermal expansion behavior.

As shown in Figure 5, the simulation results of the effective coefficient of thermal expansion in y-direction for DTH metamaterials at different length ratio λ are fixed θ = 30°. As shown in Figure 5a, when the length ratio λ = 1/10, the DTH metamaterial exhibits thermal expansion in the y-direction upon the ambient temperature rising to 60 °C, indicating positive thermal expansion behavior. As shown in Figure 5b, when the length ratio λ = 4/10, the DTH metamaterial shows almost no expansion or contraction in the y-direction under the same temperature increase, with $\alpha_{y}$ = 0.4919 ppm/°C, indicating near-zero thermal expansion behavior. As shown in Figure 5c, when the length ratio λ = 7/10, the DTH metamaterial exhibits thermal contraction in the y-direction upon the temperature rising to 60 °C, indicating negative thermal expansion behavior. Therefore, it is evident that with the angle θ  fixed, the effective thermal expansion coefficient $\alpha_{y}$ of the DTH metamaterial in the y-direction can also be tuned from positive to negative values by adjusting the length ratio λ.

**Figure 6:**
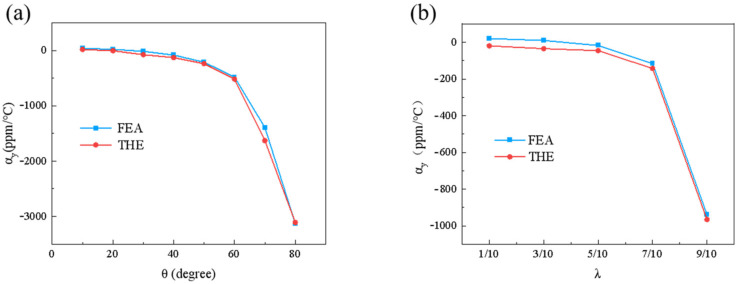
A comparison between theoretical analysis and numerical simulation results of the effective CTE ($\alpha_{y}$) in the y-direction. (**a**) The influence of different angles θ  on $\alpha_{y}$ with λ = 1/2 fixed. (**b**) The influence of different λ on $\alpha_{y}\;$ with θ = 30° fixed. The theoretical calculations fit well with the numerical results, proving the correctness of the theoretical model.

The theoretical results were compared with the numerical simulation results, as shown in Figure 6. The two scenarios of fixing λ = 1/2 and θ = 30° were validated consecutively. The results from Figure 6a,b demonstrate a strong consistency between the established theoretical model and the numerical simulation results for the CTE, thereby confirming the accuracy of the theoretical model. This validation shows that the double-trapezoidal hexagonal metamaterial (DTH) indeed exhibits tunable negative and zero thermal expansion characteristics over a wide range. This is attributed to the composition of the DTH metamaterial, which is made up of two different materials. The lower base beam $L_{1}$, made of Nylon with a high CTE, elongates more than the other beams upon thermal expansion, leading to a contraction in the height direction of the metamaterial DTH. As shown in Figure 6a, for the double-trapezoidal hexagonal metamaterial (DTH), the greater the angle θ, the more pronounced the negative CTE characteristics become. This is because as the angle θ increases, the waist beam structure, composed of PVA which has a low thermal expansion coefficient, becomes more pronounced, leading to more significant thermal mismatch effects.

Similarly, as shown in Figure 6b, increasing the length ratio λ, which also increases the length of $L_{3}$, increases the thermal expansion difference between the upper and lower bottom beams, thereby increasing the magnitude of the negative CTE of the DTH metamaterial. Clearly, the angle θ and the length ratio λ play crucial roles in tuning the negative CTE of the material. By appropriately adjusting θ and λ, the thermal expansion coefficient can be modulated. However, in practical applications, the manufacturability and consistency of these geometric parameters must be carefully considered. Although advanced manufacturing technologies, such as high-resolution 3D printing and precision microfabrication, enable precise control of θ and λ, manufacturing tolerances in large-scale production may introduce performance variations. Additionally, microstructural defects and environmental factors could further affect the material’s stability. Therefore, to ensure the reliability of DTH metamaterials in engineering applications, it is essential to optimize fabrication processes and implement stringent quality control measures to minimize parameter deviations and their impact on performance consistency.

**Figure 7:**
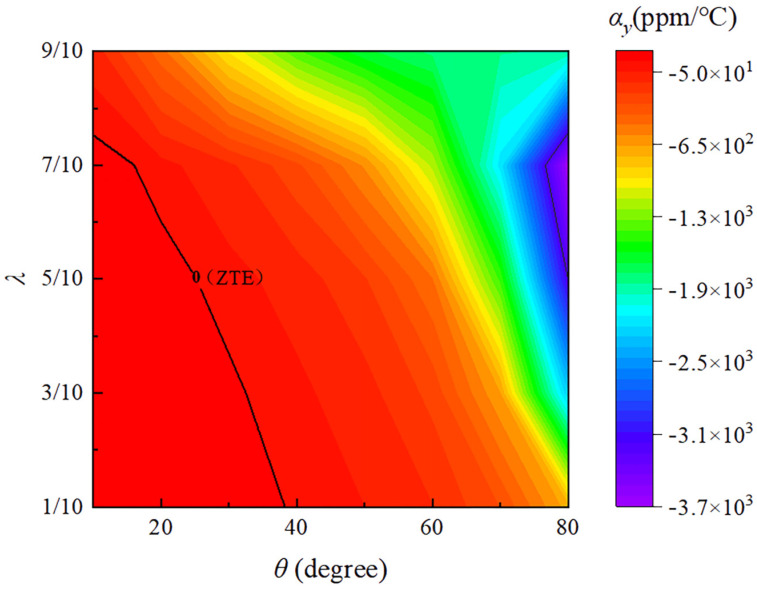
The relationship between the effective thermal expansion coefficient in the y-direction ($\alpha_{y}$) and the parameters λ and θ. The zero CTE is marked with a black line, delineating the boundaries between positive CTE (to the left of the line) and negative CTE (to the right of the line).

As shown in Figure 7 is represented as the relationship between the y-direction effective thermal expansion coefficient $\alpha_{y}$ and the parameters λ and θ. The zero CTE is marked with a black line, delineating the boundaries between positive CTE (to the left of the line) and negative CTE (to the right of the line). It can be observed that the maximum negative thermal expansion value occurs at λ = 7/10 and θ = 80°, where $\alpha_{y}$ is −3640.62 ppm/°C. The results demonstrate that even though the constituent materials of the double-trapezoidal hexagonal metamaterial (DTH), Nylon and PVA, both have positive thermal expansion coefficients, it is still possible to flexibly achieve zero CTE and a wide range of negative CTE. By adjusting the geometric parameters λ and θ, the effective CTE in the y-direction of the DTH metamaterial can be tuned to positive, zero, or even negative values, thus enabling the design of metamaterials with adjustable thermal expansion coefficients.

### 3.3. Elastic Modulus

**Figure 8:**
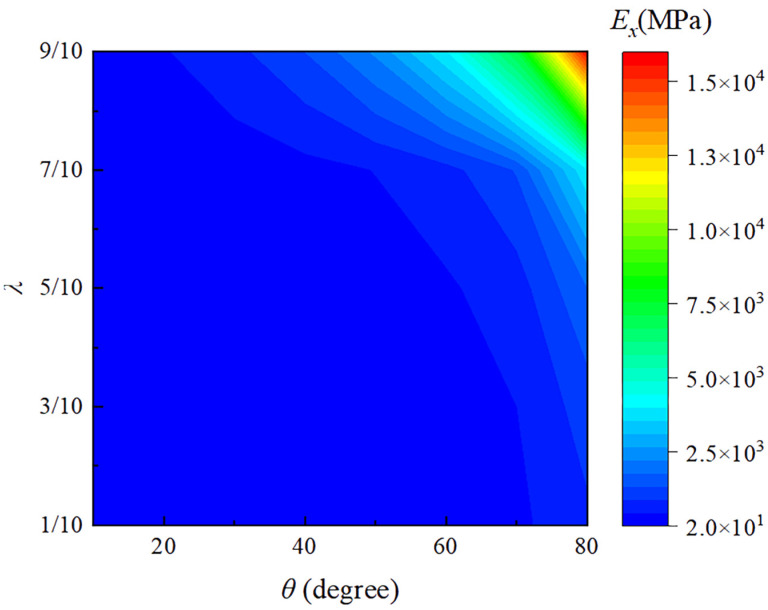
The relationship between the effective elastic moduli $E_{x}$ and the parameters λ and θ.

**Figure 9:**
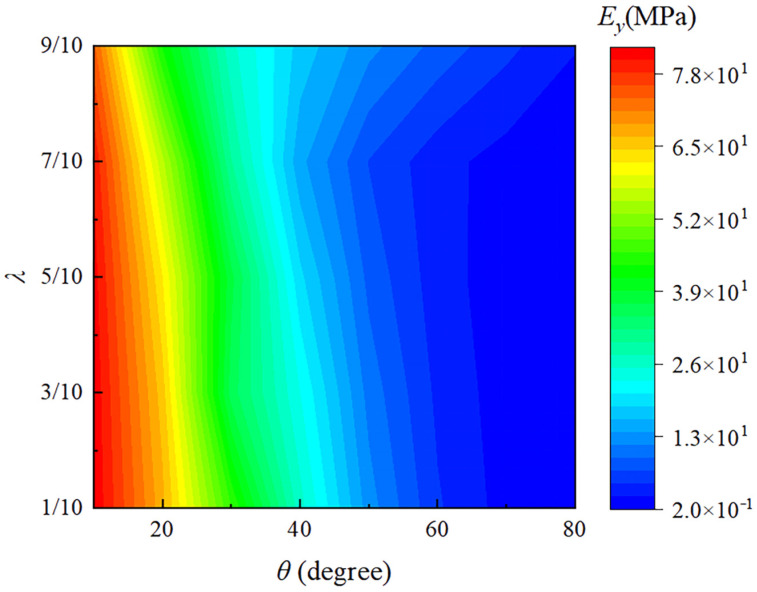
The relationship between the effective elastic moduli $E_{y}$ and the parameters λ and θ.

The elastic modulus is a crucial indicator used to analyze the resistance of materials to elastic deformation, holding significant importance in research. Generally, the stiffness of a beam is proportional to its elastic modulus. Furthermore, the stiffness of adjustable thermal expansion mechanical metamaterials is critical for practical engineering applications. Therefore, to study the stiffness of the proposed DTH metamaterial, Abaqus/CAE 2023 (Dassault Systèmes, version 2023, Providence, RI, USA) will be used next to perform uniaxial tension tests on the structure in both the x and y directions. It is important to note that when the structure is subjected only to external forces without any temperature variation, the effective elastic modulus can be determined using Equations (14) and (15) [36]. The effects of the angle θ and the length ratio λ on the effective elastic modulus are analyzed in Figure 8 and Figure 9, respectively.

(14) Ex=RFxH∆uL ,

(15) Ey=RFyL∆vH  ,

where RFx is the reaction force in the horizontal direction and ∆*u* is the horizontal displacement, RFy is the reaction force in the vertical direction and ∆*v* is the vertical displacement, H is the initial height and L is the initial length.

As shown in Figure 8 and Figure 9, the effects of different angles *θ* and length ratios *λ* on the elastic moduli $E_{x}$ and $E_{y}$ are presented, respectively. From Figure 8, it can be seen that $E_{x}$ increases with increasing θ and λ. This is because as θ increases, the overall shape of the structure becomes wider and more spread out, which relatively increases the moment of inertia in the horizontal direction. This makes the structure more resistant to deformation in the horizontal direction, thereby increasing the elastic modulus in the x-direction and enhancing its resistance to bending and deformation. Similarly, as λ increases, which means the upper base becomes longer, the trapezoidal shape more closely approximates a rectangle, increasing the horizontal moment of inertia and enhancing the structure’s bending stiffness. As shown in Figure 9, $E_{y}$ decreases as θ increases and decreases slowly with an increase in λ. The increase in θ (the angle of inclination of the side beams) makes it easier for the metamaterial structure to deform in the vertical direction, thus weakening its ability to resist deformation in this direction. Conversely, a decrease in λ increases the effective elastic modulus $E_{y}$ in the y-direction, because reducing λ causes the trapezoidal cell structure to approximate a more stable triangular configuration.

From Figure 8 and Figure 9, it can also be easily observed that the angle θ has a more significant impact on the elastic modulus $E_{y}$ in the y-direction. Overall, the changes in the effective elastic moduli $E_{x}$ and $E_{y}$ are coupled: as θ and λ increase, more load is transferred to the horizontal direction, thereby enhancing its resistance to deformation. Concurrently, this transfer reduces the support in the vertical direction, thus decreasing its resistance to vertical deformation. This coupling effect is the fundamental reason behind the opposite variations observed in the effective elastic moduli $E_{x}$ and $E_{y}$. Therefore, the main direction of force can be considered to appropriately select application scenarios, for example, high-temperature environments for shock absorption and buffering structures. The negative thermal expansion in the vertical direction absorbs thermal shocks while its lower elastic modulus provides flexible cushioning. The higher horizontal elastic modulus offers stable support; this would be beneficial in high-precision connectors in temperature-varying environments. The negative thermal expansion in the vertical direction compensates for dimensional changes due to temperature variations, while the high elastic modulus in the horizontal direction ensures connection stability. Thus, combining the adjustable thermal expansion characteristics and lightweight properties, this metamaterial can be extensively applied in various engineering fields such as aerospace, energy equipment, precision instruments, and high-temperature machinery. This versatility highlights the potential of the metamaterial to be customized for specific structural and functional requirements in demanding engineering applications.

## 4. Conclusions

This study presents a novel dual-functional metamaterial, DTH, which not only exhibits lightweight properties but also offers a broader tunable CTE range, thereby providing enhanced adjustability of its effective thermal expansion performance. Through parametric analysis and multi-scale modeling, we systematically investigated the decoupling relationships between three key performance indicators (thermal expansion coefficient, relative density, and elastic modulus) and the structural geometric parameters. The main conclusions are as follows:

(1) This study presents a dual-step hexagonal mechanical metamaterial, DTH, composed of Nylon and PVA. Its negative thermal expansion (NTE) properties arise from the thermal expansion mismatch between the two materials. The key structural parameters include the angle θ  and the length ratio λ. By adjusting θ and λ, it is possible to achieve customizable thermal expansion behavior while maintaining lightweight characteristics.
(2) Through a combination of theoretical analysis and numerical simulations, this study demonstrates that by appropriately adjusting θ and λ, the CTE of the DTH metamaterial can be tuned over a wide range, from +39.92 ppm/°C to −3640.6191 ppm/°C. This allows for the achievement of ZTE and a broader tunable range for NTE.
(3) Through finite element analysis of the correlation between the relative density of the DTH metamaterial and its geometric parameters, it is observed that within optimal parameter ranges, by decreasing λ and θ, the relative density of the DTH metamaterial gradually decreases. The relative density of DTH is significantly less than 2%, demonstrating excellent lightweight properties.
(4) Finite element analysis reveals a coupling relationship between the elastic modulus of the DTH metamaterial and its geometric parameters, allowing for precise control of its mechanical properties. This makes the material particularly suitable for engineering applications that require directional stiffness control.

The lightweight mechanical metamaterial with tunable thermal expansion proposed in this study features a simple geometric design, making it suitable for sample fabrication using additive manufacturing techniques (3D printing), thereby enhancing its feasibility for practical engineering applications. This multifunctional mechanical metamaterial effectively addresses key challenges in aerospace engineering and precision instrumentation, particularly in systems that require simultaneous lightweight design, thermal stability, and directional stiffness control. It overcomes the limitations imposed by the trade-off between thermal and mechanical properties in traditional materials, providing a revolutionary solution for next-generation space thermal protection systems and precision optical platforms. The zero thermal expansion property of the DTH metamaterial is particularly suitable for structures that require long-term geometric stability, such as satellite optical mirror frames and antenna reflector supports, while its tunable negative thermal expansion properties can be applied to spacecraft skins and protective layers to improve structural stability. Additionally, the material’s lightweight and low thermal expansion characteristics make it highly applicable in high-precision optical systems and precision manufacturing equipment, helping to reduce the impact of temperature variations on accuracy and enhancing the stability and reliability of the system. This material offers an innovative approach for next-generation devices that require high performance across multiple physical domains.

## Figures and Tables

**Table 1:** Physical properties of four materials.

Material	Density (kg/m^3^)	Elastic Modulus (MPa)	Poisson Ratio	CTE (ppm/°C)
Nylon	1150	928	0.34	102
PVA	1310	1460	0.32	42

## Data Availability

The original contributions presented in this study are included in the article. Further inquiries can be directed to the corresponding author.

## Appendix A

The local stiffness matrix of the element is as follows:

(A1) $${\left[k\right]}^{e}=\left[\begin{array}{cccccc} \frac{EA}{L} & 0 & 0 & -\frac{EA}{L} & 0 & 0\\ 0 & \frac{12EI}{L^{3}} & \frac{6EI}{L^{2}} & 0 & -\frac{12EI}{L^{3}} & \frac{6EI}{L^{2}}\\ 0 & \frac{6EI}{L^{2}} & \frac{4EI}{L} & 0 & -\frac{6EI}{L^{2}} & \frac{2EI}{L}\\ -\frac{EA}{L} & 0 & 0 & \frac{EA}{L} & 0 & 0\\ 0 & -\frac{12EI}{L^{3}} & -\frac{6EI}{L^{2}} & 0 & \frac{12EI}{L^{3}} & -\frac{6EI}{L^{2}}\\ 0 & \frac{6EI}{L^{2}} & \frac{2EI}{L} & 0 & -\frac{6EI}{L^{2}} & \frac{4EI}{L} \end{array}\right],$$

where *A* is the cross-sectional area, *E* is the elastic modulus, *I* is the moment of inertia, and *L* is the length of the beam element in the local coordinate system, as shown in Figure 2a.

The coordinate transformation matrix is as follows:

(A2) $$\left[h\right]=\left[\begin{array}{cccccc} \cos\xi & \sin\xi & 0 & 0 & 0 & 0\\ -\sin\xi & \cos\xi & 0 & 0 & 0 & 0\\ 0 & 0 & 1 & 0 & 0 & 0\\ 0 & 0 & 0 & \cos\xi & \sin\xi & 0\\ 0 & 0 & 0 & -\sin\xi & \cos\xi & 0\\ 0 & 0 & 0 & 0 & 0 & 1 \end{array}\right],$$

where ξ is the angle between the local coordinate system’s *x*-axis and the global coordinate system’s *X*-axis, measured in the clockwise direction.

The stiffness matrix of Element 1 is as follows:

(A3) $${\left[K\right]}_{1}^{e}=\left[\begin{array}{cccccc} \frac{192E_{2}I_{2}}{{\left(L_{1}\lambda \right)}^{4}} & 0 & 0 & -\frac{192E_{2}I_{2}}{{\left(L_{1}\lambda \right)}^{4}} & 0 & 0\\ 0 & \frac{4A_{2}E_{2}}{{\left(L_{1}\lambda \right)}^{2}} & 0 & 0 & -\frac{4A_{2}E_{2}}{{\left(L_{1}\lambda \right)}^{2}} & 0\\ 0 & 0 & \frac{48E_{2}I_{2}}{{\left(L_{1}\lambda \right)}^{3}} & 0 & 0 & -\frac{48E_{2}I_{2}}{{\left(L_{1}\lambda \right)}^{3}}\\ -\frac{192E_{2}I_{2}}{{\left(L_{1}\lambda \right)}^{4}} & 0 & 0 & \frac{192E_{2}I_{2}}{{\left(L_{1}\lambda \right)}^{4}} & 0 & 0\\ 0 & -\frac{4A_{2}E_{2}}{{\left(L_{1}\lambda \right)}^{2}} & 0 & 0 & \frac{4A_{2}E_{2}}{{\left(L_{1}\lambda \right)}^{2}} & 0\\ 0 & 0 & -\frac{48E_{2}I_{2}}{{\left(L_{1}\lambda \right)}^{3}} & 0 & 0 & \frac{48E_{2}I_{2}}{{\left(L_{1}\lambda \right)}^{3}} \end{array}\right]\;\;,$$

The stiffness matrix of Element 2 is as follows:

(A4) $${\left[K\right]}_{2}^{e}=\;\left[\begin{array}{cccccc} \frac{4E_{2}\left(A_{2}L_{1}^{2}{\left(1-\lambda \right)}^{2}{cos}^{2}\theta +48I_{2}{sin}^{4}\theta \right){sin}^{2}\theta }{L_{1}^{4}{\left(1-\lambda \right)}^{4}} & \frac{4E_{2}\left(A_{2}L_{1}^{2}{\left(1-\lambda \right)}^{2}-48I_{2}{sin}^{2}\theta \right){sin}^{3}\theta \cos\theta }{L_{1}^{4}{\left(1-\lambda \right)}^{4}} & 0 & -\frac{4E_{2}\left(A_{2}L_{1}^{2}{\left(1-\lambda \right)}^{2}{cos}^{2}\theta +48I_{2}{sin}^{4}\theta \right){sin}^{2}\theta }{L_{1}^{4}{\left(1-\lambda \right)}^{4}} & \frac{4E_{2}\left({-A}_{2}L_{1}^{2}{\left(1-\lambda \right)}^{2}+48I_{2}{sin}^{2}\theta \right){sin}^{3}\theta \cos\theta }{L_{1}^{4}{\left(1-\lambda \right)}^{4}} & 0\\ \frac{4E_{2}\left(A_{2}L_{1}^{2}{\left(1-\lambda \right)}^{2}-48I_{2}{sin}^{2}\theta \right){sin}^{3}\theta \cos\theta }{L_{1}^{4}{\left(1-\lambda \right)}^{4}} & \frac{4E_{2}\left(A_{2}L_{1}^{2}{\left(1-\lambda \right)}^{2}+48I_{2}{cos}^{2}\theta \right){sin}^{4}\theta }{L_{1}^{4}{\left(1-\lambda \right)}^{4}} & 0 & \frac{4E_{2}\left({-A}_{2}L_{1}^{2}{\left(1-\lambda \right)}^{2}+48I_{2}{sin}^{2}\theta \right){sin}^{3}\theta \cos\theta }{L_{1}^{4}{\left(1-\lambda \right)}^{4}} & -\frac{4E_{2}\left(A_{2}L_{1}^{2}{\left(1-\lambda \right)}^{2}+48I_{2}{cos}^{2}\theta \right){sin}^{4}\theta }{L_{1}^{4}{\left(1-\lambda \right)}^{4}} & 0\\ 0 & 0 & \frac{48E_{2}I_{2}{sin}^{3}\theta }{L_{1}^{3}{\left(1-\lambda \right)}^{3}} & 0 & 0 & -\frac{48E_{2}I_{2}{sin}^{3}\theta }{L_{1}^{3}{\left(1-\lambda \right)}^{3}}\\ -\frac{4E_{2}\left(A_{2}L_{1}^{2}{\left(1-\lambda \right)}^{2}{cos}^{2}\theta +48I_{2}{sin}^{4}\theta \right){sin}^{2}\theta }{L_{1}^{4}{\left(1-\lambda \right)}^{4}} & \frac{4E_{2}\left({-A}_{2}L_{1}^{2}{\left(1-\lambda \right)}^{2}+48I_{2}{sin}^{2}\theta \right){sin}^{3}\theta \cos\theta }{L_{1}^{4}{\left(1-\lambda \right)}^{4}} & 0 & \frac{4E_{2}\left(A_{2}L_{1}^{2}{\left(1-\lambda \right)}^{2}{cos}^{2}\theta +48I_{2}{sin}^{4}\theta \right){sin}^{2}\theta }{L_{1}^{4}{\left(1-\lambda \right)}^{4}} & \frac{4E_{2}\left(A_{2}L_{1}^{2}{\left(1-\lambda \right)}^{2}-48I_{2}{sin}^{2}\theta \right){sin}^{3}\theta \cos\theta }{L_{1}^{4}{\left(1-\lambda \right)}^{4}} & 0\\ \frac{4E_{2}\left({-A}_{2}L_{1}^{2}{\left(1-\lambda \right)}^{2}+48I_{2}{sin}^{2}\theta \right){sin}^{3}\theta \cos\theta }{L_{1}^{4}{\left(1-\lambda \right)}^{4}} & -\frac{4E_{2}\left(A_{2}L_{1}^{2}{\left(1-\lambda \right)}^{2}+48I_{2}{cos}^{2}\theta \right){sin}^{4}\theta }{L_{1}^{4}{\left(1-\lambda \right)}^{4}} & 0 & \frac{4E_{2}\left(A_{2}L_{1}^{2}{\left(1-\lambda \right)}^{2}-48I_{2}{sin}^{2}\theta \right){sin}^{3}\theta \cos\theta }{L_{1}^{4}{\left(1-\lambda \right)}^{4}} & \frac{4E_{2}\left(A_{2}L_{1}^{2}{\left(1-\lambda \right)}^{2}+48I_{2}{cos}^{2}\theta \right){sin}^{4}\theta }{L_{1}^{4}{\left(1-\lambda \right)}^{4}} & 0\\ 0 & 0 & -\frac{48E_{2}I_{2}{sin}^{3}\theta }{L_{1}^{3}{\left(1-\lambda \right)}^{3}} & 0 & 0 & \frac{48E_{2}I_{2}{sin}^{3}\theta }{L_{1}^{3}{\left(1-\lambda \right)}^{3}} \end{array}\right]\;,$$

The stiffness matrix of Element 3 is as follows:

(A5) $${\left[K\right]}_{3}^{e}=\left[\begin{array}{cccccc} \frac{192E_{1}I_{1}}{L_{1}^{4}} & 0 & 0 & -\frac{192E_{1}I_{1}}{L_{1}^{4}} & 0 & 0\\ 0 & \frac{4A_{1}E_{1}}{L_{1}^{2}} & 0 & 0 & -\frac{4A_{1}E_{1}}{L_{1}^{2}} & 0\\ 0 & 0 & \frac{48E_{1}I_{1}}{L_{1}^{3}} & 0 & 0 & -\frac{48E_{1}I_{1}}{L_{1}^{3}}\\ -\frac{192E_{1}I_{1}}{L_{1}^{4}} & 0 & 0 & \frac{192E_{1}I_{1}}{L_{1}^{4}} & 0 & 0\\ 0 & -\frac{4A_{1}E_{1}}{L_{1}^{2}} & 0 & 0 & \frac{4A_{1}E_{1}}{L_{1}^{2}} & 0\\ 0 & 0 & -\frac{48E_{1}I_{1}}{L_{1}^{3}} & 0 & 0 & \frac{48E_{1}I_{1}}{L_{1}^{3}} \end{array}\right]\;,$$

The assembly of the global stiffness matrix is as follows:

(A6) $$\left[K\right]=\left[\begin{array}{ccccccccc} K_{11} & K_{12} & K_{13} & K_{14} & K_{15} & K_{16} & K_{17} & K_{18} & K_{19}\\ K_{21} & K_{22} & K_{23} & K_{24} & K_{25} & K_{26} & K_{27} & K_{28} & K_{29}\\ K_{31} & K_{32} & K_{33} & K_{34} & K_{35} & K_{36} & K_{37} & K_{38} & K_{39}\\ K_{41} & K_{42} & K_{43} & K_{44} & K_{45} & K_{46} & K_{47} & K_{48} & K_{49}\\ K_{51} & K_{52} & K_{53} & K_{54} & K_{55} & K_{56} & K_{57} & K_{58} & K_{59}\\ K_{61} & K_{62} & K_{63} & K_{64} & K_{65} & K_{66} & K_{67} & K_{68} & K_{69}\\ K_{71} & K_{72} & K_{73} & K_{74} & K_{75} & K_{76} & K_{77} & K_{78} & K_{79}\\ K_{81} & K_{82} & K_{83} & K_{84} & K_{85} & K_{86} & K_{87} & K_{88} & K_{89}\\ K_{91} & K_{92} & K_{93} & K_{94} & K_{95} & K_{96} & K_{97} & K_{98} & K_{99} \end{array}\right]\;\;,$$

The global thermal load is given as follows:

(A7) $$\left\{P\right\}=\left\{\begin{array}{c} A_{2}∆TE_{2}\alpha \\ 0\\ 0\\ A_{2}E_{2}\alpha ∆T\left(\sin\theta -1\right)\\ -A_{2}∆TE_{2}\alpha \cos\theta \\ 0\\ -\alpha ∆T\left(A_{1}E_{1}+A_{2}E_{2}\sin\theta \right)\\ A_{2}∆TE_{2}\alpha \cos\theta \\ 0 \end{array}\right\}\;,$$

Simplified thermoelastic model:

(A8) $$\left\{\begin{array}{c} P_{{\bar{v}}_{1}}\\ P_{{\bar{u}}_{2}}\\ P_{{\bar{v}}_{2}}\\ M_{{\bar{\varphi }}_{2}}\\ P_{{\bar{u}}_{3}} \end{array}\right\}=\left[\begin{array}{ccccc} K_{11} & K_{12} & K_{13} & K_{14} & K_{15}\\ K_{21} & K_{22} & K_{23} & K_{24} & K_{25}\\ K_{31} & K_{32} & K_{33} & K_{34} & K_{35}\\ K_{41} & K_{42} & K_{43} & K_{44} & K_{45}\\ K_{51} & K_{52} & K_{53} & K_{54} & K_{55} \end{array}\right]\left\{\begin{array}{c} {\bar{v}}_{1}\\ {\bar{u}}_{2}\\ {\bar{v}}_{2}\\ {\bar{\varphi }}_{2}\\ {\bar{u}}_{3} \end{array}\right\}-\left\{\begin{array}{c} {K_{T}}_{11}\\ {K_{T}}_{21}\\ {K_{T}}_{31}\\ {K_{T}}_{41}\\ {K_{T}}_{51} \end{array}\right\}\;\;,$$

where

(A9) $$K_{11}=\frac{4A_{2}E_{2}}{{\left(L_{1}\lambda \right)}^{2}}\;\;,$$

(A10) $$K_{21}=K_{12}=0,$$

(A11) $$K_{31}=-\frac{4A_{2}E_{2}}{{\left(L_{1}\lambda \right)}^{2}},$$

(A12) $$K_{41}=K_{42}=K_{51}=0,$$

(A13) $$K_{22}=\frac{A_{2}E_{2}\left(1-\cos\left(4\theta \right)\right)}{2L_{1}^{2}{\left(1-\lambda \right)}^{2}}+\frac{{192E}_{2}I_{2}{sin}^{6}\theta }{L_{1}^{4}{\left(1-\lambda \right)}^{4}}+\frac{{192E}_{2}I_{2}}{{\left(L_{1}\lambda \right)}^{4}},$$

(A14) $$K_{32}=K_{23}=\frac{4E_{2}\left(A_{2}L_{1}^{2}{\left(1-\lambda \right)}^{2}-48I_{2}{sin}^{2}\theta \right){sin}^{3}\theta \cos\theta }{L_{1}^{4}{\left(1-\lambda \right)}^{4}},$$

(A15) $$K_{52}=K_{25}=-\frac{4E_{2}\left(A_{2}L_{1}^{2}{\left(1-\lambda \right)}^{2}{cos}^{2}\theta +48I_{2}{sin}^{4}\theta \right){sin}^{2}\theta }{L_{1}^{4}{\left(1-\lambda \right)}^{4}},$$

(A16) $$K_{13}=-\frac{4A_{2}E_{2}}{{\left(L_{1}\lambda \right)}^{2}},$$

(A17) $$K_{33}=\frac{4A_{2}E_{2}{sin}^{4}\theta }{L_{1}^{2}{\left(1-\lambda \right)}^{2}}+\frac{4A_{2}E_{2}}{{\left(L_{1}\lambda \right)}^{2}}+\frac{192E_{2}I_{2}{sin}^{4}\theta {cos}^{2}\theta }{L_{1}^{4}{\left(1-\lambda \right)}^{4}}\;,$$

(A18) $$K_{43}=0,$$

(A19) $$K_{53}=K_{35}=\frac{4E_{2}\left({-A}_{2}L_{1}^{2}{\left(1-\lambda \right)}^{2}+48I_{2}{sin}^{2}\theta \right){sin}^{3}\theta \cos\theta }{L_{1}^{4}{\left(1-\lambda \right)}^{4}},$$

(A20) $$K_{14}=K_{24}=K_{34}=K_{54}=0,$$

(A21) $$K_{44}=\frac{48E_{2}I_{2}{sin}^{3}\theta }{L_{1}^{3}{\left(1-\lambda \right)}^{3}}+\frac{48E_{2}I_{2}}{{\left(L_{1}\lambda \right)}^{3}},$$

(A22) $$K_{15}=K_{45}=0\;,$$

(A23) $$K_{55}=\frac{A_{2}E_{2}\left(1-\cos\left(4\theta \right)\right)}{2L_{1}^{2}{\left(1-\lambda \right)}^{2}}+\frac{{192E}_{1}I_{1}}{L_{1}^{4}}+\frac{{192E}_{2}I_{2}{sin}^{6}\theta }{L_{1}^{4}{\left(1-\lambda \right)}^{4}},$$

(A24) $$\;\;\;\;\;\;\;\;\;\;\;\;\;\;\left\{\begin{array}{c} {K_{T}}_{11}\\ {K_{T}}_{21}\\ {K_{T}}_{31}\\ {K_{T}}_{41}\\ {K_{T}}_{51} \end{array}\right\}=\left\{\begin{array}{c} 0\\ A_{2}E_{2}\alpha ∆T\left(\sin\theta -1\right)\\ -A_{2}∆TE_{2}\alpha \cos\theta \\ 0\\ -\alpha ∆T\left(A_{1}E_{1}+A_{2}E_{2}\sin\theta \right) \end{array}\right\}$$
